# Pilot Study of Home-Based Virtual Reality Fitness Training in Post-Discharge Rehabilitation for Patients with Spinal Cord Injury: A Randomized Double-Blind Multicenter Trial

**DOI:** 10.3390/life14070859

**Published:** 2024-07-09

**Authors:** Dongheon Kang, Seon-Deok Eun, Jiyoung Park

**Affiliations:** 1 Department of Healthcare and Public Health Research, National Rehabilitation Center, Ministry of Health and Welfare, Seoul 01022, Republic of Korea; jakekang@korea.kr; 2 Department of Safety and Health, Wonkwang University, Iksan 54538, Republic of Korea

**Keywords:** spinal cord injuries, clinical trial protocol, intervention, virtual reality, physical fitness

## Abstract

Spinal cord injury (SCI) patients require continuous rehabilitation post-discharge to ensure optimal recovery. This study investigates the effectiveness of home-based virtual reality fitness training (VRFT) as a convenient and accessible rehabilitation method for SCI patients. This randomized, double-blind, multicenter trial will enroll 120 participants, assigning them to either an 8-week VRFT program (exercise group) or a control group engaging in regular daily activities. The outcomes measured include muscle function, cardiopulmonary fitness, body composition, and physical performance. Our study will determine the safety and feasibility of VRFT in a home setting for SCI patients and evaluate whether these patients can effectively participate in such a program post-discharge. The results of this study are expected to inform future exercise protocols for SCI rehabilitation, offering valuable insights into the utility of VRFT as a therapeutic tool.

## 1. Introduction

Spinal cord injury (SCI) patients face various deficiencies due to the extent of their injuries and disabilities [1,2]. This population experiences severe physical impairments and substantial psychological and social consequences, affecting their quality of life [3,4]. The prevalence of SCI is significant, with approximately 2500 new cases reported annually in South Korea [5].

Many SCI patients endure prolonged hospital stays for initial treatment and rehabilitation. During this period, the central nervous system exhibits heightened neuroplasticity, facilitating potential functional improvements [6,7,8,9]. Early and intensive rehabilitation interventions have been instrumental in enhancing outcomes for SCI patients, reducing morbidity and improving overall quality of life. Nevertheless, managing SCI patients post-discharge remains a critical challenge to ensure continued recovery and successful reintegration into the community [3,4,8,9,10]. The efficacy of rehabilitation is heavily influenced by the intensity and specificity of the therapy, with repetitive, task-oriented, and intensive therapies being the most effective [11,12]. Therefore, continuous and intensive treatment is crucial for SCI patients to achieve optimal recovery [13].

Typically, SCI patients are offered outpatient therapy post-discharge. However, attending these sessions is often challenging due to long travel distances, inclement weather situations, and transportation problems [13]. Furthermore, due to high demand, patients often face long waiting periods and are given a restricted number of sessions.

SCI patients present a wide range of rehabilitation needs due to the extent of their injuries and the resulting disabilities. The diverse deficiencies include motor and sensory impairments, autonomic dysfunction, and secondary conditions such as pressure sores, spasticity, and chronic pain. Training conducted at home has the potential to significantly enhance access to rehabilitation for SCI patients, allowing them to extend their training period and potentially achieve better outcomes [13]. According to rehabilitation guidelines, individuals should take part in independent training, focusing on daily targets to guide their efforts [14,15].

Simulated training, such as virtual reality training, leverages computer systems to monitor user movements, facilitating interaction with on-screen games or activities. This technology ensures a reliable, user-friendly, and engaging recovery process for SCI patients, free from usage constraints [16,17,18]. In particular, virtual reality fitness training (VRFT) has the advantage of enabling participants to exercise through a game-based program that uses their physical abilities in real time [18]. Various research has demonstrated that VRFT positively impacts several physical health domains, including muscle function, cardiopulmonary fitness, body composition, and physical performance in SCI patients. Studies have shown that VRFT matches or surpasses the effectiveness of conventional therapy [16,17,19,20,21,22]. By providing interactive and integrated sensory game experiences that engage the entire body, VRFT serves as a substitute for conventional training [23]. This variation significantly alleviates the repetitiveness of conventional therapy, making workouts more fun. Implementing game-based techniques and immersive environments has shown promise in motivating individuals with SCI to participate in more rehabilitation training after being discharged from the hospital [23]. This optimistic outlook on the effectiveness of these methods is encouraging for the future of SCI rehabilitation.

This approach promotes participants’ engagement in vigorous physical activities by minimizing the dependency on assistant intervention. Thus, VRFT serves as a valuable addition or substitute to conventional therapy programs, potentially significantly enhancing patients’ quality of life after hospital discharge.

A variety of studies have examined the effectiveness of home-centered VRFT in enhancing fitness variables and rehabilitation post-SCI, indicating the feasibility of VRFT platforms for ongoing in-home recovery [18,24,25,26,27]. Nevertheless, patients with SCI involved in home-centered VRFT programs require well-defined methods to assess their functional capacity, identify physical limitations, and enhance their health condition and progress after hospital discharge. Various studies have indicated that individuals found home-centered VRFT satisfactory, although the specifics of program implementation can differ widely [18,23,24,27,28]. Barriers to using home-based VRFT include insufficient technological experience among users and technical issues [24].

Home-centered VRFT has the potential to serve as an addition or substitute to conventional rehabilitation programs for patients with SCI, with the possibility of significantly enhancing their quality of life [17,19,20,21,23]. Consequently, VRFT platforms appear promising for facilitating rehabilitation at home.

Game-centered VRFT helps to alleviate the boredom often associated with traditional exercise therapy by incorporating captivating and sensory elements. Virtual reality training provides an engaging and effective way to maintain physical activity by involving users in interactive settings and promoting exercises that engage the whole body [23,25]. Using gamified techniques and immersive environments, individuals with SCI show greater encouragement to participate in a workout [23,26]. Consequently, home-based VRFT minimizes the need for direct intervention, fosters vigorous physical activity, and increases engagement more effectively than in hospital environments [27,28].

The American Spinal Injury Association encourages patients with SCI to improve their physical health, emphasizing muscle function, physical performance, cardiopulmonary fitness, and body composition [28]. Nevertheless, numerous individuals with SCI residing in the community after hospital discharge are involved in activities that lack sufficient physical activity or do not effectively support functional recovery [11]. Moreover, the engagement of individuals with SCI in house-centered exercises is restricted, highlighting the need for more secure exercise options [14]. One novel approach involves home-centered VRFT, supported by a physician’s prescription, to guarantee patient safety during exercise sessions.

SCI patients who have transitioned from hospital-based rehabilitation to the community should be evaluated on the effectiveness of a VRFT customized to their health condition based on their condition and medical prescription. Nevertheless, to date, the Republic of Korea lacks research examining the safety of house-centered VRFT participation for individuals with SCI discharged from hospital when guided by a physician’s prescription. Therefore, our research group, comprising experts in exercise and rehabilitation medicine, intends to examine the impact of house-centered VRFT on individuals with SCI following discharge from hospital, under the guidance of a physician’s prescription.

## 2. Methods

### 2.1. Experimental Design

This trial is structured as a multicenter, randomized, double-blind, parallel group that focuses on individuals with their first spinal cord injury. Random assignment of individuals to the exercise or control group will be allocated with evaluators unaware of group placements during subsequent assessments.

The study design ensures that all participants receive the standard care appropriate for their condition. The control group will continue their usual daily routines without additional exercise interventions, reflecting a real-world scenario where not all patients may have access to or engage in structured rehabilitation programs. This design allows us to evaluate the additional benefits of the VRFT program. Any participant identified as needing further rehabilitation will be referred for appropriate care, ensuring no one is deprived of necessary medical support.

The trial will be conducted at four distinct hospitals across South Korea: (1) National Rehabilitation Center and Hospital, (2) Bucheon SM Hospital and Sports Center, (3) Goyang Rehabilitation Sports Center and Korea National Health Insurance Service Hospital in Ilsan, and (4) Goodplayground Center and Korea University Anam Hospital.

Eligible participants will be enrolled in the spinal cord injury rehabilitation programs at the involved hospitals, which cater to both inpatients and outpatients. During the study, an investigator will evaluate with the participants’ consent and remain unaware of their group assignments.

**Figure 1:**
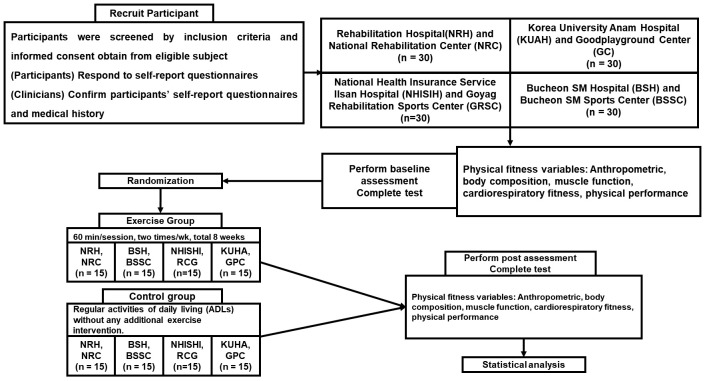
Flowchart. NRH, National Rehabilitation Hospital; NRC, National Rehabilitation Center; BSH, Bucheon SM Hospital; BSSC, Bucheon SM Sports Center; NHISHI, Korea National Health Insurance Service Hospital in Ilsan; RCG, Rehabilitation–sports Center in Goyang; KUHA, Korea University Hospital in Anam; GPC, Good Playground Center.

The research protocol will commence under the direction of the National Rehabilitation Hospital. This study received approval from the Institutional Review Board at the National Rehabilitation Hospital (National Rehabilitation Ceter-43-5-2021). Registration of the study protocol was completed with the identifier KCT0007521. This protocol adheres to the SPIRIT guidelines [29]. A flowchart outlining the study design is illustrated in Figure 1.

### 2.2. Participants

Participants may be eligible for the study if they (1) have been diagnosed with SCI, (2) have completed their initial hospitalization and rehabilitation, and are now living in the community, and (3) have provided informed consent and are willing to participate in this research. To address ethical concerns, all participants, including those in the control group, will continue to receive standard care and support as per clinical guidelines. Those requiring immediate or additional rehabilitation will not be excluded from receiving necessary interventions outside the scope of the study. Participants will not be eligible for the study if they (1) are presently receiving inpatient care, (2) cannot participate in exercise activities because of other nervous system disorders (apart from SCI), lower limb orthopedic issues, or severe cardiovascular or respiratory conditions, and (3) are assessed by the evaluator as unable to perform the required tasks.

### 2.3. Randomization

Following the acquisition of informed consent from the participants, each team supervisor will identify and refer suitable candidates for the study. Subsequently, an independent statistician, not affiliated with the study team, will conduct the randomization process. Within each institution, participants will be categorized into two distinct subgroups through a block randomization approach. By employing blocks of size two in each stratum, an equal distribution ratio of 1:1 between the experimental and control groups will be achieved.

The randomization schedule will be generated utilizing SPSS software version 23.0 (IBM Corp., Armonk, NY, USA). The statistician responsible for the randomization will determine the block size and seed number through a random selection process. Thereafter, the randomized allocations will be sealed in packets, each designated for a participant, and delivered to the manager when the trial begins.

### 2.4. Random Assignment

The research implements a double-blind methodology ensuring that neither the individuals involved nor the assessors know the allocation of the groups. To maintain blinding, independent assessors who are unaware of the group assignments will conduct all evaluations. Additionally, participants will be instructed not to discuss their specific activities with the assessors or other participants. Trainers administering the interventions will be kept unaware of the study details to minimize possible prejudice to the integrity of outcomes, thus strengthening the research results’ validity. Nonetheless, it should be noted that the principal investigator conducting the study will be aware of the group assignments due to the nature of the trial. Participants in the control group will adhere to their usual daily routines without any additional exercise intervention. They will continue with their regular activities of daily living (ADLs) as they did before the study, without any structured or guided exercise program.

### 2.5. Protocol

At the initial visit, the study team will evaluate potential participants to determine if they meet the inclusion or exclusion criteria. The investigators will provide detailed explanations of the study and ensure that informed consent is obtained by presenting consent forms and discussing the study with the participants. This stage involves obtaining documented approval from all participants. Subsequently, individuals with SCI fill out a guided survey and submit it to a medical professional for medical status and assess the responses. Prior to randomization, qualified assessors will evaluate the outcome variables for each participant.

### 2.6. Program

The training program consists of 1 h classes conducted biweekly over a period of 2 months, as detailed in Table 1. Participants in the exercise group will have access to the VRFT devices not only during the biweekly classes but also at home throughout the trial period. This will allow the participants to engage in additional sessions as desired.

**Figure 2:**
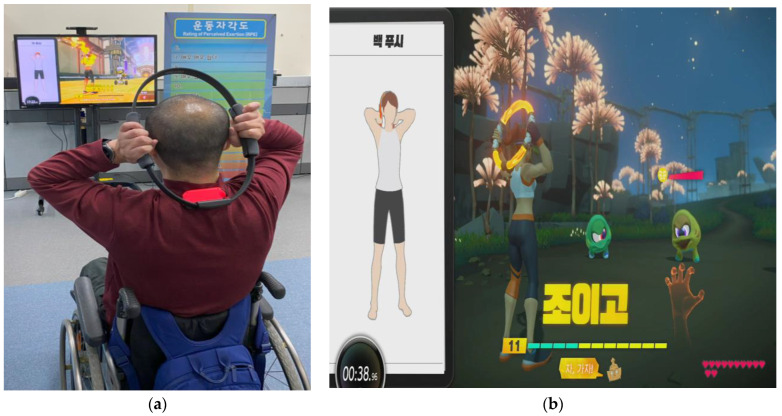
Intervention program. (**a**) Experimental group—participant interacting with a virtual reality fitness training apparatus. (**b**) Experimental group—home-based use of the virtual reality fitness training system.

The rehabilitation protocol for spinal cord injury comprised cardiovascular and strength training exercises, as well as practical exercises focusing on the extremities and core. Furthermore, specialized programs were designed considering the goals and the type of exercise, time, frequency, duration, and intensity, adhering to the guidelines of the American College of Sports Medicine. The intervention program will incorporate the use of the Ringfit Adventure (RA) game for the Nintendo Switch (NS). (Figure 2). RA, a fitness program for the Nintendo, utilizes semi-immersive technology.

Individuals with SCI will require an NS system, a Ring-Con (RC) (a flexible exercise ring held by the participant), a Bluetooth operator (attached to the RC and the other fastened to a leg strap on the participant’s quadriceps), and a monitor for this training class. The class is scheduled twice a week over eight weeks. Each class will be 1 h long, consisting of 10 min for warming up, 10 min for cooling down, and 40 min dedicated to the primary exercise session. RA is an action role-playing fitness game that advances the storyline through the player’s physical activities, which directly influence the character’s on-screen movements. These exercises are performed with the aid of the RC and thigh band [30]. The RC contains highly accurate pressure and tension detectors that capture and translate the individual’s movements [31]. Additionally, the Bluetooth operator features an infrared motion sensor capable of tracking the participant’s pulse rate [32]. RA has the capability to assess the ideal intensity of physical activities tailored to each individual and modify it according to biological signals either lowering or raising the activity level. In the first class, the virtual coach determines the intensity of physical activities and then progressively modifies them according to feedback from later sessions. The research staff will consistently supervise individuals in the exercise group, who will encourage participants to sustain the 16-session class at 60% to 80% of the individual’s baseline and the highest heart rate. Participants will be guided to keep their exertion within the range of 4 to 8 on the modified Borg CR10 rating of perceived exertion scale, which translates to a perception of effort from ‘moderate’ to ‘vigorous’ [33]. Participants in the comparison group will adhere to their normal routines and will not receive any specific intervention throughout the study.

### 2.7. Measures

Individuals with SCI will perform initial assessments and subsequent evaluations after 16 classes, utilizing the assessment outlined (Table 2). A custom-designed questionnaire will be used to gather descriptive and sociodemographic information. Outcomes will measure muscle function (including grip strength), cardiopulmonary fitness (including VO_2_peak and respiratory function), body composition (through full-body DXA scanning and bioimpedance measurement), and physical performance (functional reach test) to determine overall physical fitness. The primary outcome of this study is physical fitness factors, specifically muscle strength, cardiopulmonary fitness, body composition, and physical performance. Secondary outcomes include psychological factors such as quality of life. Individuals with SCI will perform initial assessments and subsequent evaluations after 16 classes utilizing the assessment outlined in Table 2. Incorporating these spinal cord injury-specific measures will provide a thorough evaluation of the participants’ rehabilitation progress and functional gains following the training.

#### 2.7.1. Muscle Function

The upper extremity muscle strength assessment will be conducted by measuring the grip strength of each participant using a hand dynamometer (TKK-5401; Takei Scientific Instruments, Tokyo, Japan). Participants will be positioned with their shoulders gently drawn inward and in a neutral rotation, the elbow at a 90-degree angle, and the forearm and wrist maintained in a neutral posture. Each hand will undergo three testing sessions, preceded by a practice trial. The mean value of the three recorded measurements will be utilized for each hand [34].

#### 2.7.2. Cardiopulmonary Fitness

A progressive peak oxygen uptake test will be administered to all participants to assess their cardiopulmonary fitness. Using an arm crank ergometer (Lode), participants will start at a workload of 10 W for 2 min. The workload will increase by 10 W every 2 min until the participant reaches voluntary exhaustion. The peak aerobic power will be defined as the VO_2_ measurement when the participant can no longer maintain a cadence of 60 rotations per minute (RPM) [35]. A nose-and-mouth facemask will be used to collect expired air, which will be analyzed for oxygen uptake and carbon dioxide output every 10 s using a gas exchange system (K5 system, Cosmed, Rome, Italy). A Polar heart rate monitor will continuously monitor the heart rate, and the rating of perceived exertion (RPE) will be recorded using the Borg scale.

The assessment of respiratory function will be conducted using a digital spirometer (Pony FX, COSMED, Rome, Italy) [36]. Participants will receive thorough explanations and demonstrations of the test procedures to ensure accurate measurements, after which the respiratory function tests will be conducted [37]. This study will measure parameters such as forced vital capacity (FVC), vital capacity (VC), peak expiratory flow (PEF), forced expiratory volume in 1 s (FEV1), inspiratory reserve volume (IRV), expiratory reserve volume (ERV), and inspiratory capacity (IC). The evaluation of respiratory muscle strength will involve assessing the maximum expiratory pressure (MEP) and maximum inspiratory pressure (MIP) using the Pony FX device (COSMED, Rome, Italy) [37].

#### 2.7.3. Physical Performance

The Functional Reach Test (FRT) will be utilized to assess physical performance [38]. The procedure will involve participants sitting upright in a wheelchair and extending both arms forward as far as possible. The initial measurement point will be the position of the fingertips when the arms are raised to shoulder height. The final measurement point will be the furthest distance reached, maintained for 3–5 s. The distance between these two points (in cm) will be recorded. An evaluator will assist from the side. Each participant will perform three attempts, and the average of these attempts will be used for analysis.

#### 2.7.4. Body Composition

Body composition will be assessed using DXA (Discovery Wi, Hologic, Marlborough, MA, USA) and bioimpedance testing (InbodyS10, Inbody, Seoul, Republic of Korea). DXA will provide extensive details on lean and fat masses for different regions of the body, whereas BIA will evaluate multiple body composition indicators, including BMI, percentage of body fat, muscle mass, and overall weight.

#### 2.7.5. Quality of Life

The World Health Organization Quality of Life (WHOQOL-BREF) questionnaire will be administered to assess the participants’ quality of life. This comprehensive assessment tool evaluates multiple dimensions of an individual’s overall well-being. The questionnaire comprises 26 items, each rated on a 5-point Likert scale. The participants will be required to rate their responses based on their experiences over the past two weeks.

### 2.8. Participant Timeline

The schedule for recruiting, conducting experiments, and evaluating study subjects is detailed in the participant timeline, as outlined by the SPIRIT guidelines in Table 3 [29].

### 2.9. Data Collection Methods and Analysis

Outcome measures will be collected before and after the 16 intervention sessions, excluding the sociodemographic and descriptive information.

The sample size determination was based on prior research findings [34]. A power analysis was conducted using G*Power software (version 3.1.2.) [39]. The analysis used an effect size of 0.5 for the outcomes, a power level of 80% (type II error of 0.2), and a significance level of 0.05. We calculated that 29 individuals per group would be required to achieve the necessary statistical power. Therefore, we plan to enroll 30 individuals per group, resulting in a total of 120 participants across the four groups. This calculation ensures sufficient power to detect meaningful differences between the intervention and control groups.

We will assess participant characteristics and baseline comparisons to assess the comparability between the exercise and control groups. A table will be provided to display the characteristics of individuals assigned by the group. Percentages and frequencies will be used to present categorical elements, and time intervals along with continuous elements will be summarized using medians, standard deviations, means, and interquartile ranges. This procedure secures the baseline equivalence between the groups.

An intention-to-treat framework will be employed for our statistical analysis. Kolmogorov–Smirnov analysis will be used to assess the normality of the dependent variables for the outcomes. The responsible variables will be characterized based on their distribution, utilizing either medians and interquartile ranges or means and standard deviations. To achieve the objective, hypothesis tests will be conducted for each outcome, positing that the exercise group will have superior outcomes compared to the control group. All estimates will be accompanied by intervals with 95% confidence level. Student’s *t*-test will be applied for independent samples that follow a normal distribution, while the Mann–Whitney U test will be utilized for data that do not conform to a normal distribution.

## 3. Discussion

This multicenter, randomized, double-blind study represents a pioneering effort to evaluate the efficacy of house-centered VRFT in individuals with SCI released from medical care with a physician’s recommendation in South Korea. Optimal SCI care recommends that rehabilitation be administered in the individuals’ residences or local environments according to the specific requirements and choices [40]. Rehabilitation exercises performed at home are enabled through VRFT, an innovative modality [41]. VRFT platforms are compact, easy to use, and allow clinicians to monitor them remotely, making them particularly suited for use at home [42]. VRFT conducted at home is appropriate for individuals with SCI released from intensive hospital treatment and seeking to restore their regular functions. Following hospital-based rehabilitation, this is crucial for individuals with advanced SCI to either sustain or intensify their therapy regimen. VRFT acts as an adjunct therapy to conventional face-to-face rehabilitation, usually taking place one to three times weekly. This approach enables the continuation of therapeutic exercises after formal rehabilitation has concluded. Prior research indicates that an increase of at least 15 h in rehabilitative exercise is necessary to significantly improve post-SCI functional recovery [43]. Individuals with SCI might find it simpler to meet the necessary physical activity requirements if they have the flexibility to carry out daily workouts at home according to their own plan.

VRFT conducted at home has the capability to increase workout intensity, promote rehabilitation, and enhance conditioning. Our research serves as an initial step towards validating this hypothesis. To date, only a limited number of small randomized controlled trials have investigated VRFT in a residential environment. The previous study utilized a motion-sensing exercise regimen conducted three times a week in a domestic setting, supplemented by twice-weekly clinic visits [44]. This research will utilize a VRFT platform offering a diverse array of exercises, thereby enabling enhanced personalization to achieve the specific therapeutic objectives of SCI patients. Moreover, the VRFT outlined in this study will be conducted completely within the home environment.

We expect that VRFT will be deemed practical, given that the devices can be easily set up in participants’ residences and that individuals can efficiently master and advance with VRFT. Additionally, it is anticipated that individuals will find VRFT enjoyable and appreciate its advantages in their rehabilitation journey, avoiding negative outcomes like accidents or mishaps.

The results from this study will offer a framework for individuals with SCI to securely engage in VRFT and workout at home after hospital-based rehabilitation. Our plan is to present our results in academic publications and share them at symposia, distributing the information through meetings focused on SCI rehabilitation.

It is essential to recognize that this study will have limitations due to the small number of participants, with just 15 individuals assigned per exercise group. Undertaking a pilot study is a sensible initial step before investing substantial resources in the study; however, the limited sample size might restrict our capacity to identify meaningful variations among the groups. Following studies, our study plans to undertake an extensive study to assess the impact of VRFT conducted at home on physical outcomes, including muscle function, cardiopulmonary fitness, body composition, and functional abilities. The findings will guide future studies by providing insights into aspects such as physical measurements, sample size, VRFT duration, weekly frequency, exercise intensity, time, and type of interventions. Practical insights into home-based VRFT post-hospital discharge are essential for guiding future study endeavors. Our study focuses on how hospital-based professionals and fitness trainers can work together to facilitate the implementation of VRFT for individuals with SCI after their hospital discharge. We aim to make substantial progress in this area over the next few years. The increasing adoption of technology in home-based settings positions VRFT as a promising method to enhance rehabilitation intensity and improve health conditions for those recuperating for individuals with SCI.

## 4. Conclusions

This study is poised to provide novel insights into the efficacy of VRFT programs for individuals with SCI, facilitating their safe engagement in home-based VRFT under the supervision of a healthcare professional post-discharge. We also seek to explore the feasibility of home-based exercise for SCI patients following their discharge from the hospital. Additionally, should this innovative exercise training method demonstrate effectiveness, it could generate significant data to inform the creation of more thorough rehabilitation programs for individuals with SCI moving forward. Additionally, should this innovative exercise training method demonstrate effectiveness, it could generate significant data to inform the creation of more thorough rehabilitation programs for individuals with SCI moving forward.

## Figures and Tables

**Table 1:** Intervention protocol.

Warm-Up/Cool-Down		Upper Extremities	Trunk
Aerobic	Flexibility		
Freewheeling	- Static stretching - Dynamic stretching	- Back press - Overhead press - Front press - Bow pull - Shoulder press - Triceps kickback - Overhead arm-twist - Overhead arm spin	- Overhead side bend - Overhead twist - Pendulum bend - Overhead bend - Seated forward press - Seated twist - Russian twist - Seated ring raise - Ring raise combo - Fan pose - Warrior pose - Standing forward fold - Hinge pose

**Table 2:** Collection of data: medical information and anthropometric measurement part data.

Medical Part	Age	Years
	Gender	Male or female
	American Spinal Injury Association (ASIA) level	A, B, C, D, E
	Hypertension, anemia, dyspnea or asthma, orthostatic hypotension, diabetes, medications for heart disease, coronary stent, epilepsy, medications for anticoagulants, medications for depression, rigidity, autonomic dysreflexia, bladder management, bowel care, pressure ulcer, acute low back pain within 4 weeks, joint pain, medications for osteoporosis, hip or femur fractures	Yes or no
Anthropometric Part	Blood pressure	mmHg
	Height	cm
	Weight	kg
	Body mass index	Underweight/normal weight/overweight
	Days of discharge from the hospital	Number of days

**Table 3:** Duration of study: timeline for enrollment, interventions, and assessments in this study.

Study Period				
	Enrollment	Allocation	Post-Allocation	Closeout
Timepoint, baseline	t	0	T16	tx
Enrollment				
Eligibility screening	X			
Informed consent	X			
(Participant) Self-report questionnaires	X			
(Clinicians) Confirm participants’ self-report questionnaires and medical history and write doctor’s notes	X			
Allocation		X		
Assessments				
Baseline variables		X		
Post-intervention variables			X	X
Intervention				
Experimental group			X	X

## Data Availability

The authors will make the data available upon reasonable request.

## References

[1] Singh A., Tetreault L., Kalsi-Ryan S., Nouri A., Fehlings M.G. (2014). Global prevalence and incidence of traumatic spinal cord injury. Clin. Epidemiol..

[2] Lee B.B., Cripps R.A., Fitzharris M., Wing P.C. (2014). The global map for traumatic spinal cord injury epidemiology: Update 2011, global incidence rate. Spinal Cord.

[3] Furlan J.C., Sakakibara B.M., Miller W.C., Krassioukov A.V. (2013). Global incidence and prevalence of traumatic spinal cord injury. Can. J. Neurol. Sci..

[4] New P.W., Sundararajan V. (2008). Incidence of non-traumatic spinal cord injury in Victoria, Australia: A population-based study and literature review. Spinal Cord.

[5] Shin H. (2020). Etiology and epidemiology of spinal cord injury in Korea. J. Korean Med. Assoc..

[6] Curt A., Van Hedel H.J., Klaus D., Dietz V. (2008). Recovery from a spinal cord injury: Significance of compensation, neural plasticity, and repair. J. Neurotrauma.

[7] Dietz V., Fouad K. (2014). Restoration of sensorimotor functions after spinal cord injury. Brain.

[8] Elbasiouny S.M., Moroz D., Bakr M.M., Mushahwar V.K. (2010). Management of spasticity after spinal cord injury: Current techniques and future directions. Neurorehabil. Neural Repair..

[9] Fehlings M.G., Tetreault L.A., Wilson J.R., Kwon B.K., Burns A.S., Martin A.R., Hawryluk G., Harrop J.S. (2017). A clinical practice guideline for the management of acute spinal cord injury: Introduction, rationale, and scope. Glob. Spine J..

[10] Kirshblum S.C., Burns S.P., Biering-Sørensen F., Donovan W., Graves D.E., Jha A., Waring W. (2011). International standards for neurological classification of spinal cord injury (revised 2011). J. Spinal Cord Med..

[11] Hicks A.L., Martin K.A., Ditor D.S., Latimer A.E., Maher J.L., McCartney N., Phillips S.M. (2003). Long-term exercise training in persons with spinal cord injury: Effects on strength, arm ergometry performance and psychological well-being. Spinal Cord.

[12] Van Hedel H.J.A., Dietz V. (2010). Rehabilitation of locomotion after spinal cord injury. Restor. Neurol. Neurosci..

[13] Dallmeijer A.J., van der Woude L.H. (2001). Health related functional status in men with spinal cord injury: Relationship with lesion level and endurance capacity. Spinal Cord.

[14] Nooijen C.F., Stam H.J., Sluis T., Valent L., Twisk J., Van Den Berg-Emons R.J. (2017). A behavioral intervention promoting physical activity in people with subacute spinal cord injury: Secondary effects on health, social participation and quality of life. Clin. Rehabil..

[15] Latimer A.E., Martin Ginis K.A., Arbour K.P. (2006). The efficacy of an implementation intention intervention for promoting physical activity among individuals with spinal cord injury: A randomized controlled trial. Rehabil. Psychol..

[16] De Araújo A.V.L., Neiva J.F.D.O., Monteiro C.B.D.M., Magalhães F.H. (2019). Efficacy of virtual reality rehabilitation after spinal cord injury: A systematic review. BioMed Res. Int..

[17] Asadzadeh A., Samad-Soltani T., Salahzadeh Z., Rezaei-Hachesu P. (2021). Effectiveness of virtual reality-based exercise therapy in rehabilitation: A scoping review. Inform. Med. Unlocked.

[18] Vibhuti, Kumar N., Kataria C. (2023). Efficacy assessment of virtual reality therapy for neuromotor rehabilitation in home environment: A systematic review. Disabil. Rehabil. Assist. Technol..

[19] De Miguel-Rubio A., Rubio M.D., Alba-Rueda A., Salazar A., Moral-Munoz J.A., Lucena-Anton D. (2020). Virtual reality systems for upper limb motor function recovery in patients with spinal cord injury: Systematic review and meta-analysis. JMIR mHealth uHealth.

[20] Costa M.T.S., Vieira L.P., de Oliveira Barbosa E., Oliveira L.M., Maillot P., Vaghetti C.A.O., Monteiro-Junior R.S. (2019). Virtual reality-based exercise with exergames as medicine in different contexts: A short review. Clin. Pract. Epidemiol. Ment. Health.

[21] Rutkowski S., Kiper P., Cacciante L., Cieslik B., Mazurek J., Turolla A., Szczepanska-Gieracha J. (2020). Use of virtual reality-based training in different fields of rehabilitation: A systematic review and meta-analysis. J. Rehabil. Med..

[22] Kang D., Park J., Eun S.D. (2023). Home-based virtual reality exergame program after stroke rehabilitation for patients with stroke: A study protocol for a multicenter, randomized controlled trial. Life.

[23] De Miguel-Rubio A., Rubio M.D., Salazar A., Camacho R., Lucena-Anton D. (2020). Effectiveness of virtual reality on functional performance after spinal cord injury: A systematic review and meta-analysis of randomized controlled trials. J. Clin. Med..

[24] Sweet S.N., Rocchi M., Arbour-Nicitopoulos K., Kairy D., Fillion B. (2017). A telerehabilitation approach to enhance quality of life through exercise among adults with paraplegia: Study protocol. JMIR Res. Protoc..

[25] Touchett H., Apodaca C., Siddiqui S., Huang D., Helmer D.A., Lindsay J.A., Skelton F. (2022). Current approaches in telehealth and telerehabilitation for spinal cord injury (TeleSCI). Curr. Phys. Med. Rehabil. Rep..

[26] Chemtob K., Rocchi M., Arbour-Nicitopoulos K., Kairy D., Fillion B., Sweet S.N. (2019). Using tele-health to enhance motivation, leisure time physical activity, and quality of life in adults with spinal cord injury: A self-determination theory-based pilot randomized control trial. Psychol. Sport Exerc..

[27] Solomon R.M., Dhakal R., Halpin S.J., Hariharan R., O’Connor R.J., Allsop M., Sivan M. (2022). Telerehabilitation for individuals with spinal cord injury in low-and middle-income countries: A systematic review of the literature. Spinal Cord.

[28] Rupp R., Biering-Sørensen F., Burns S.P., Graves D.E., Guest J., Jones L., Kirshblum S. (2021). International standards for neurological classification of spinal cord injury: Revised 2019. Top. Spinal Cord Inj. Rehabil..

[29] Chan A.W., Tetzlaff J.M., Altman D.G., Laupacis A., Gøtzsche P.C., Krleža-Jerić K., Hróbjartsson A., Mann H., Dickersin K., Berlin J.A. (2013). SPIRIT 2013 statement: Defining standard protocol items for clinical trials. Ann. Intern. Med..

[30] Koivisto J., Hamari J. (2019). The rise of motivational information systems: A review of gamification research. Int. J. Inf. Manag..

[31] Tuan S.H., Chang L.H., Sun S.F., Lin K.L., Tsai Y.J. (2022). Using exergame-based exercise to prevent and postpone the loss of muscle mass, muscle strength, cognition, and functional performance among elders in rural long-term care facilities: A protocol for a randomized controlled trial. Front. Med..

[32] Lu C., Hassan L., Buruk O., Nummenmaa T., Peltonen J. ‘Switch’ up your exercise: An empirical analysis of online user discussion of the ring fit adventure exergame. Proceedings of the 5th International GamiFIN Conference.

[33] Williams N. (2017). The Borg Rating of Perceived Exertion (RPE) scale. Occup. Med..

[34] Adams J., Lai B., Rimmer J., Powell D., Yarar-Fisher C., Oster R.A., Fisher G. (2022). Telehealth high-intensity interval exercise and cardiometabolic health in spinal cord injury. Trials.

[35] El-Sayed M.S., Younesian A. (2005). Lipid profiles are influenced by arm cranking exercise and training in individuals with spinal cord injury. Spinal Cord.

[36] Park J., Kang D., Eun S.D. (2021). Development and pilot testing of novel game-based respiratory rehabilitation exercise devices for patients with tetraplegia. Technol. Health Care.

[37] Kang D., Park J., Eun S.D. (2022). A preliminary study on the feasibility of community game-based respiratory muscle training for individuals with high cervical spinal cord injury levels: A novel approach. BMC Sports Sci. Med. Rehabil..

[38] Arsh A., Darain H., Rahman M.U., Ullah I., Shakil-Ur-Rehman S. (2021). Reliability of modified functional reach test in the assessment of balance function in people with spinal cord injury: A systematic review. JPMA J. Pak. Med. Assoc..

[39] Faul F., Erdfelder E., Lang A.G., Buchner A.G. (2007). G*Power 3: A flexible statistical power analysis program for the social, behavioral, and biomedical sciences. Behav. Res. Methods.

[40] Harkema S., MacKay-Lyons M. (2013). Best practices for the management of individuals with spinal cord injury. J. Rehabil. Res. Dev..

[41] Van Schaik P., Blake J., Pernet F., Spears I., Fencott C. (2008). Virtual augmented exercise gaming for older adults. Cyberpsychol. Behav..

[42] Sarupuri B., Kulpa R., Aristidou A., Multon F. (2024). Dancing in virtual reality as an inclusive platform for social and physical fitness activities: A survey. Vis. Comput..

[43] Tharu N.S., Wong A.Y.L., Zheng Y.P. (2023). Neuromodulation for recovery of trunk and sitting functions following spinal cord injury: A comprehensive review of the literature. Bioelectron. Med..

[44] Holden M.K. (2005). Virtual environments for motor rehabilitation. Cyberpsychol. Behav..

